# Imaging Characteristics for Predicting Cognitive Impairment in Patients With Cerebral Autosomal Dominant Arteriopathy With Subcortical Infarcts and Leukoencephalopathy

**DOI:** 10.3389/fnagi.2022.876437

**Published:** 2022-06-10

**Authors:** Akira Taniguchi, Akihiro Shindo, Ken-ichi Tabei, Osamu Onodera, Yukio Ando, Takao Urabe, Kazumi Kimura, Kazuo Kitagawa, Yoshihiro Miyamoto, Misa Takegami, Masafumi Ihara, Ikuko Mizuta, Toshiki Mizuno, Hidekazu Tomimoto

**Affiliations:** ^1^Department of Neurology, Mie University Graduate School of Medicine, Tsu, Japan; ^2^School of Industrial Technology, Advanced Institute of Industrial Technology, Tokyo Metropolitan Public University Corporation, Tokyo, Japan; ^3^Department of Neurology, Clinical Neuroscience Branch, Brain Research Institute, Niigata University, Niigata, Japan; ^4^Department of Neurology, Graduate School of Medical Sciences, Kumamoto University, Kumamoto, Japan; ^5^Department of Amyloidosis Research, Nagasaki International University, Nagasaki, Japan; ^6^Department of Neurology, Juntendo University Urayasu Hospital, Chiba, Japan; ^7^Department of Neurology, Graduate School of Medicine, Nippon Medical School, Tokyo, Japan; ^8^Department of Neurology, Tokyo Women’s Medical University, Tokyo, Japan; ^9^Open Innovation Center, National Cerebral and Cardiovascular Center, Osaka, Japan; ^10^Department of Preventive Medicine and Epidemiology, National Cerebral and Cardiovascular Center, Osaka, Japan; ^11^Department of Neurology, National Cerebral and Cardiovascular Center, Suita, Japan; ^12^Department of Neurology, Graduate School of Medical Science, Kyoto Prefectural University of Medicine, Kyoto, Japan

**Keywords:** small vessel disease, CADASIL, dementia, cognitive impairment, lacunar, white matter, microbleeds

## Abstract

**Objectives:**

Patients with cerebral autosomal dominant arteriopathy with subcortical infarcts and leukoencephalopathy (CADASIL) show various clinical symptoms, including migraine, recurrent stroke, and cognitive impairment. We investigated the associations between magnetic resonance imaging (MRI) markers of small vessel disease and neuropsychological tests and identified the MRI characteristics for predicting cognitive impairment in patients with CADASIL.

**Methods:**

Subjects included 60 CADASIL patients diagnosed with genetic tests and registered in the Japanese CADASIL REDCap database between June 2016 and December 2020. Patient information including clinical data, modified Rankin Scale (mRS); MRI findings of small vessel disease including periventricular and deep white matter lesions (WML), lacunar infarcts, and cerebral microbleeds (CMBs); and neuropsychological tests, including the Japanese version of the Mini-Mental State Examination (MMSE), the Japanese version of the Montreal Cognitive Assessment (MoCA-J), and the Frontal Assessment Battery (FAB), were evaluated.

**Results:**

Data from 44 CADASIL patients were eligible for this study, compared between patients with and without dementia. Regarding the neuroimaging findings, the Fazekas score of periventricular and deep WML was higher in patients with dementia (periventricular, *p* = 0.003; deep, *p* = 0.009). The number of lacunar infarcts was higher in patients with dementia (*p* = 0.001). The standardized partial regression coefficient (SPRC) in MoCA-J was 0.826 (95% CI, 0.723–0.942; *p* = 0.005) for the number of CMBs. The SPRC in MMSE was 0.826 (95% CI, 0.719–0.949; *p* = 0.007) for the number of CMBs. The SPRC for FAB decreased significantly to 0.728 (95% CI, 0.551–0.960; *p* = 0.024) for the number of lacunar infarcts. Receiver operating characteristic (ROC) curves for dementia showed that in the number of lacunar infarcts, a cut-off score of 5.5 showed 90.9% sensitivity and 61.1% specificity. For the number of CMBs, a cut-off score of 18.5 showed 45.5% sensitivity and 100% specificity.

**Conclusion:**

The characteristic MRI findings were that CADASIL patients with dementia had severe WML, both periventricular and deep, and a larger number of lacunar infarcts than those without dementia. The risk of dementia may be associated with ≥ 6 lacunar infarcts, ≥19 CMBs, or a Fazekas scale score of 3 in periventricular and deep WML.

## Introduction

The term cerebral small vessel disease (SVD) is used with various meanings and contexts, including pathological, clinical, and neuroimaging aspects (Pantoni, 2010). There are different types of SVD, and the most common forms are arteriolosclerosis and cerebral amyloid angiopathy (Pantoni, 2010). Characteristics of magnetic resonance imaging (MRI) with SVD include lacunar infarcts, white matter lesions (WML), cerebral microbleeds (CMBs), and enlarged perivascular spaces (ePVS) (Pantoni, 2010; Wardlaw et al., 2013, 2019). These characteristic MRI appearances are associated with a decline in cognitive function in patients with SVD (Wardlaw et al., 2019); however, it remains unclear how each type of SVD lesion induces cognitive decline in the absence of Alzheimer’s pathology.

Cerebral autosomal dominant arteriopathy with subcortical infarcts and leukoencephalopathy (CADASIL) is an autosomal dominant disease and inherited type of SVD (Pantoni, 2010). CADASIL is also a common pure form of subcortical vascular dementia (Roman et al., 2002). Mutations in the *NOTCH3* gene, which were identified in 1996, cause of granular osmiophilic material deposition in the small arteries, the major component of which is Notch3 ectodomain, resulting in the progressive degeneration of vascular smooth muscle cells, leading to small vessel arteriopathy in the central nervous system (Joutel et al., 1996, 2000; Ueda et al., 2015). Patients with CADASIL commonly exhibit migraine with aura around the age of 30 years, subcortical ischemic events at 50 years, and cognitive impairment between 50 and 60 years (Chabriat et al., 2009). WML, extensive white matter hyperintensities in the anterior temporal lobes and multiple lacunar infarcts have been identified as representative MRI findings in CADASIL (Chabriat et al., 1998; Bersano et al., 2018). Several previous studies have shown an association between cognitive decline and MRI findings in CADASIL (Liem et al., 2009; Yoon et al., 2015; Shi et al., 2018). An increase in lacunar infarcts and CMBs can be associated with cognitive decline in patients (Liem et al., 2009). However, the cut-off points of these imaging markers with dementia risk of patients with CADASIL are still unclear. Moreover, although these MRI findings are characteristic of SVD, the total SVD score did not show a significant association with cognitive performance in patients with CADASIL (Shi et al., 2018). These results raised the question as to which SVD markers can be associated with cognitive impairment in patients with CADASIL.

Recently, we constructed a database of patients with CADASIL (Shindo et al., 2020). The present study investigated SVD imaging markers and cognitive impairment in Japanese patients with CADASIL. Moreover, we evaluated the risk of dementia, identified the possible imaging factors associated with dementia, and assessed the cut-off points of each imaging markers.

## Patients and Methods

### Patients

We constructed the REDCap database for CADASIL patients using a Research Electronic Data Capture (REDCap) system (Harris et al., 2009; Shindo et al., 2020). After obtaining consent and approval from the local institutional review board committee, the physician sent a registration form to the secretariat at the National Cerebral and Cardiovascular Center of Japan. Written consent was obtained from all patients. This study was conducted in accordance with the Declaration of Helsinki and approved by the Ethics Committee of Mie University Graduate School of Medicine (permit number 2918).

Detailed clinical information included age, sex, age at onset, height, weight, body mass index (BMI), systolic and diastolic blood pressure (BP), pulse rate, smoking habit, drinking habit, past medical history (hypertension, hyperlipidemia, diabetes mellitus, renal dysfunction, ischemic heart disease, atrial fibrillation), migraine (with or without aura), ischemic stroke, hemorrhagic stroke, type of mutation in the *NOTCH3* gene, family history, treatment, modified Rankin Scale (mRS) (Shinohara et al., 2006), findings of MRI [number of lacunar infarcts, number of CMBs, deep and periventricular Fazekas scale (Fazekas et al., 1987), major cerebral artery stenosis], and skin biopsy results. Moreover, the patients underwent neuropsychological tests, including the Japanese version of the Mini-Mental State Examination (MMSE) (Mori et al., 1985), the Japanese version of the Montreal Cognitive Assessment (MoCA-J) (Nasreddine et al., 2005; Fujiwara et al., 2010), the Frontal Assessment Battery (FAB) (Dubois et al., 2000), and the Trail Making Test (TMT) A and B.

The diagnosis of CADASIL was based on the genetic criteria that either the patient or a third-degree relative had *NOTCH3* mutations (Shindo et al., 2020). Dementia was diagnosed in each hospital, and defined according to the diagnostic criteria found in the fourth or fifth edition of the Diagnostic and Statistical Manual for Mental Disorders (DSM-IV or DSM-V).

### Statistical Analysis

Patient sex, past medical history, complete TMT-A/B tasks, and MRI findings regarding the presence of anterior temporal pole lesions, WML, lacunar infarcts, CMBs, and major cerebral artery stenosis in patients with and without dementia were analyzed using *χ^2^* tests. Age, height, weight, BMI, blood pressure, mRS, neuropsychological test results, and MRI findings of periventricular Fazekas scale, deep Fazekas scale, number of lacunar infarcts, and number of CMBs were analyzed using the Mann-Whitney *U* test. Continuous variables were summarized as median and range. Spearman’s or Pearson’s rank correlation analyses were performed to identify correlations between neuropsychological tests and neuroradiological findings. Independent variables for multiple regression analysis were input using forward selection (likelihood ratio). Receiver operating characteristic (ROC) curves were constructed to assess the sensitivity and specificity of scores in predicting dementia and MRI findings. The SPSS Statistics 24 software package was used to perform descriptive statistical analyses. Statistical significance was set at *p* < 0.05.

## Results

### Study Patients

A total of 60 patients with CADASIL were registered in 11 hospitals using the REDCap system. Of those, we excluded 16 patients as they were not assessed with neuropsychological tests. Therefore, data from 44 patients were collected and compared between patients with and without dementia.

The demographic and clinical data of the patients with and without dementia is shown in Table 1. A total number of 21 *NOTCH3* mutations was found, and a novel mutation (p.C355G) was found in two cases (Table 2). Of the 44 patients, 15 fulfilled the diagnostic criteria for dementia, and 29 did not have dementia. The mean age at onset was 58.1 years old for all patients and 26 patients were male. The mean BMI was 22.8 kg/m2. The mean systolic BP was 116.6 mmHg, mean diastolic BP was 72.8 mmHg, and mean pulse rate was 75.3 beats per minute. There was no significant difference between the groups with and without dementia considering the age, sex, age at onset, height, weight, BMI, systolic and diastolic BP, pulse rate, smoking habit, drinking habit, and past medical history. Although the mean mRS in all patients was 1.7, the mRS in patients with dementia was 2.8, which was significantly higher than that in the group without dementia (*p* < 0.001). Regarding living habits, six patients showed current smoking and 18 showed former smoking. Four patients with dementia (26.7%) were current smokers, and the rate of current smokers tended to be higher in the dementia group (*p* = 0.099). Among the neuropsychological tests, the MoCA-J, MMSE, and FAB scores were lower in patients with dementia (*p* < 0.001), and the completion rate of TMT-A/B was lower in the dementia group (set A, *p* = 0.013; set B, *p* = 0.001). Regarding the neuroimaging findings, WML was detected in all patients and anterior temporal lobe lesions were detected in 41 patients (93.2%). The Fazekas scores of periventricular and deep WML were higher in patients with dementia than in those without dementia (periventricular, *p* = 0.003; deep, *p* = 0.009). Lacunar infarcts were detected in 40 patients (90.9%), and the number was higher in patients with dementia (*p* = 0.001). CMBs were detected in 30 patients (68.2%). The total number of CMBs (*p* = 0.059), lobar CMBs (*p* = 0.084), and deep CMBs (*p* = 0.077) were higher in patients with dementia.

**TABLE 1:** Characteristics in the 44 CADASIL patients.

	All	Dementia	No dementia	*p* value
			
	*n* = 44	*n* = 15	*n* = 29	
Mean age (years)	58.1 ± 9.9	61.8 ± 10.4	56.2 ± 9.3	0.099
Male sex, (n, %)	26 (59.1%)	10 (66.7%)	16 (55.2%)	0.343
Height (cm)	165.0 ± 8.1	166.5 ± 9.7	165.0 ± 7.2	0.365
Weight (kg)	62.7 ± 10.2	62.7 ± 10.2	61.4 ± 10.1	0.123
Body mass index (kg/m^2^)	22.8 ± 2.8	23.4 ± 2.2	22.5 ± 3.1	0.273
Blood pressure and pulse rate				
Systolic blood pressure (mmHg)	116.6 ± 14.9	116.3 ± 17.7	116.9 ± 13.4	0.312
Diastolic blood pressure (mmHg)	72.8 ± 10.0	71.7 ± 9.4	73.4 ± 10.1	0.462
Pulse rate (beat per minute)	75.3 ± 12.1	77.2 ± 12.8	74.2 ± 11.8	0.528
Modified rankin scale	1.7 ± 1.3	2.8 ± 1.0	1.1 ± 0.9	<0.001*
**Living habit**				
Current smoking (n, %)	6 (13.6%)	4 (26.7%)	2 (6.9%)	0.099
Former smoking (n, %)	18 (40.9%)	6 (40.0%)	12 (41.4%)	0.559
Alcohol (n, %)	15 (34.1%)	4 (26.7%)	11 (37.9%)	0.315
**Past medical history**				
Migraine (n, %)	13 (29.5%)	2 (13.3%)	11 (37.9%)	0.086
Migraine with aura (n, %)	6 (13.6%)	1 (6.7%)	5 (17.4%)	0.320
Hypertension (n, %)	6 (13.6%)	4 (26.7%)	2 (6.9%)	0.092
Dyslipidemia (n, %)	17 (38.6%)	6 (40.0%)	11 (37.9%)	0.573
Diabetes mellitus (n, %)	0 (0.0%)	0 (0.0%)	0 (0.0%)	–
Ischemic heart disease (n, %)	0 (0.0%)	0 (0.0%)	0 (0.0%)	–
**Neuropsychological tests**				
MoCA-J score	20.4 ± 7.2	12.3 ± 7.0	23.4 ± 5.5	<0.001*
MMSE score	24.5 ± 7.0	16.9 ± 5.9	27.8 ± 5.8	<0.001*
FAB score	12.7 ± 5.0	7.6 ± 3.8	15.1 ± 4.2	<0.001*
TMT				
Complete set-A (total number = 17 n, %) **	9 (56.3%)	1 (16.7%)	8 (72.7%)	0.013*
set-A time (s)	71.4 ± 45.3	80	70.4 ± 48.3	–
Complete set-B (total number = 17, n, %) **	11 (64.7%)	1 (16.7%)	10 (90.9%)	0.001*
set-B time (s)	119.0 ± 50.8	223	99.0 ± 49.0	–
**MRI findings**				
Anterior temporal pole lesion (n, %)	41 (93.2%)	14 (93.3%)	28 (96.6%)	0.571
White matter lesion (n, %)	44 (100%)	15 (100%)	29 (100%)	–
Periventricular white matter lesion (Fazekas)	2.6 ± 0.6	2.9 ± 0.3	2.4 ± 0.7	0.003*
Deep white matter lesion (Fazekas)	2.6 ± 0.6	2.9 ± 0.3	2.5 ± 0.6	0.009*
Presence of lacunar infarcts (n, %s)	40 (90.9%)	15 (93.3%)	25 (86.2%)	0.175
Number of lacunar infarcts (n)	7.6 ± 6.9	11.8 ± 6.6	5.6 ± 6.3	0.001*
Presence of CMBs (n, %s)	30 (68.2%)	12 (80.0%)	18 (62.1%)	0.194
Total number of CMBs (n)	7.9 ± 13.0	9.0 ± 17.5	7.3 ± 10.3	0.059
Number of lobar CMBs (n)	3.0 ± 7.0	4.3 ± 9.9	2.4 ± 5.0	0.084
Number of deep CMBs (n)	4.2 ± 5.9	4.2 ± 7.4	4.2 ± 5.2	0.077
Major cerebral artery stenosis (n, %)	6 (13.6%)	3 (20.0%)	3 (10.3%)	0.327

Japanese version of the Montreal Cognitive Assessment (MoCA-J), Mini Mental State Examination (MMSE), the Frontal Assessment Battery (FAB), the Trail Making Test (TMT), and cerebral microbleeds (CMBs). *p < 0.05.

**TABLE 2:** Summary of NOTCH3 mutations.

Nucleotide change	Amino acid change	Number of cases
c.224G > C	p.R75P	2
c.277T > G	p.C93G	1
c.316T > C	p.C106R	2
c.391G > T	p.G131C	1
c.397C > T	p.R133C	4
c.421C > T	p.R141C	9
c.457C > T	p.R153C	1
c.505C > T	p.R169C	1
c.544C > T	p.R182C	5
c.554G > A	p.C185Y	1
c.635G > A	p.C212Y	2
c.665G > C	p.C222S	1
c.773A > G	p.Y258C	1
c.994C > T	p.R332C	3
*c.1063T > G	p.C355G	2
c.1163G > A	p.C388Y	1
c.1255T > C	p.C419R	2
c.1630C > T	p.R544C	1
c.1819C > T	p.R607C	2
c.2185T > G	p.C729G	1
c.3062A > G	p.Y1021C	1
		total 44

*c.1063T > G (p. C355G) identified as a novel mutation.

### Correlations Between Neuropsychological Tests and Neuroradiological Findings

The correlations between neuropsychological and neuroradiological findings are summarized in Table 3. Periventricular WML demonstrated a negative correlation with the MoCA-J (*p* = 0.016), MMSE (*p* = 0.001), and FAB scores (*p* = 0.032), and a positive correlation with the TMT-B scores (*p* = 0.035). Deep WML demonstrated a negative correlation with the MoCA-J (*p* = 0.024) and MMSE scores (*p* = 0.002). The presence of lacunar infarcts negatively correlated with the MMSE scores (*p* = 0.037), and the total number of lacunar infarcts demonstrated a negative correlation with the MoCA-J (*p* = 0.002), MMSE (*p* = 0.005), and FAB scores (*p* = 0.043), and a positive correlation with TMT-A scores (*p* = 0.001). The presence of CMBs did not correlate with the results of any of the neuropsychological tests; however, the total number of CMBs and lobar and deep CMBs negatively correlated with the MoCA-J (*p* = 0.004, 0.004, and 0.028, respectively) and MMSE scores (*p* = 0.003, 0.014, and 0.017, respectively).

**TABLE 3:** Correlations between neuropsychological tests and neuroradiological findings.

		Neuropsychological tests				
	
Neuroradiological findings		MoCA-J	MMSE	FAB	TMT-A	TMT-B
**White matter lesions**						
periventricular	Spearman ρ	−0.412	−0.538	−0.436	0.030	0.637
	*p* value	0.016*	0.001*	0.023*	0.939	0.035*
deep	Spearman ρ	−0.386	−0.494	−0.31	−0.104	0.299
	*p* value	0.024*	0.002*	0.115	0.791	0.372
**Lacunar infarcts**						
presence	Spearman ρ	−0.269	−0.345	−0.292	0.137	0.500
	*p* value	0.125	0.037*	0.140	0.725	0.117
total number of lacunar infarcts	Pearson γ	−0.507	−0.452	−0.392	0.892	0.592
	*p* value	0.002*	0.005*	0.043*	0.001*	0.055
**CMBs**						
presence	Spearman ρ	0.000	−0.164	−0.098	0.000	0.120
	*p* value	1.000	0.339	0.625	1.000	0.726
total number of CMBs	Pearson γ	−0.574	−0.577	−0.564	0.158	0.525
	*p* value	0.004*	0.003*	0.012*	0.800	0.226
number of lobar CMBs	Pearson γ	−0.351	−0.391	−0.244	0.252	0.103
	*p* value	0.004*	0.014*	0.211	0.547	0.777
number of deep CMBs	Pearson γ	−0.372	−0.381	−0.252	−0.247	−0.155
	*p* value	0.028*	0.017*	0.197	0.555	0.669
**Temporal pole lesion**						
presence	Spearman ρ	0.090	0.091	0.027		0.300
	*p* value	0.615	0.591	0.892		0.370

*p < 0.05.

### Factors Associated With Neuropsychological Tests

Factors associated with each neuropsychological test for MRI findings such as anterior temporal pole lesion, perivascular and deep WML, the number of lacunar infarcts, and CMBs are summarized in Table 4. The standardized partial regression coefficient (SPRC) in MoCA-J was 0.655 (95% CI, 0.461–0.931; *p* = 0.018) for the number of lacunar infarcts and 0.826 (95% CI, 0.723–0.942; *p* = 0.005) for the number of CMBs. The SPRC in MMSE was 0.826 (95% CI, 0.719–0.949; *p* = 0.007) for the number of CMBs. The SPRC in FAB was 0.728 (95% CI, 0.551–0.960; *p* = 0.024) for the number of lacunar infarcts. There was no significant association between these neuropsychological tests and either the anterior temporal pole lesion or WML.

**TABLE 4:** Factors associated with neuropsychological tests.

	MoCA-J			MMSE			FAB		
			
Neuroradiological findings	SPRC	95%CI	*P*	SPRC	95%CI	*P*	SPRC	95%CI	*P*
Anterior temporal pole lesion	0.619	0.000–7428	0.92	26.09	0.002–444039	0.512	4.105	0.004–3889	0.686
White matter lesion									
Periventricular white matter	0.217	0.000–349.0	0.685	0.033	0.000–75.4	0.388	0.695	0.003–138.4	0.893
Deep white matter	0.16	0.000–14978	0.754	7.889	0.000–791841	0.725	1	–	–
Number of lacunar infarcts	0.655	0.461–0.931	0.018*	0.725	0.483–1.089	0.122	0.728	0.551–0.960	0.024*
Number of CMBs	0.826	0.723–0.942	0.005*	0.826	0.719–0.949	0.007*	0.912	0.832–1.000	0.050

*p < 0.05.

### Receiver Operating Characteristic Curves for Dementia

**FIGURE 1:**
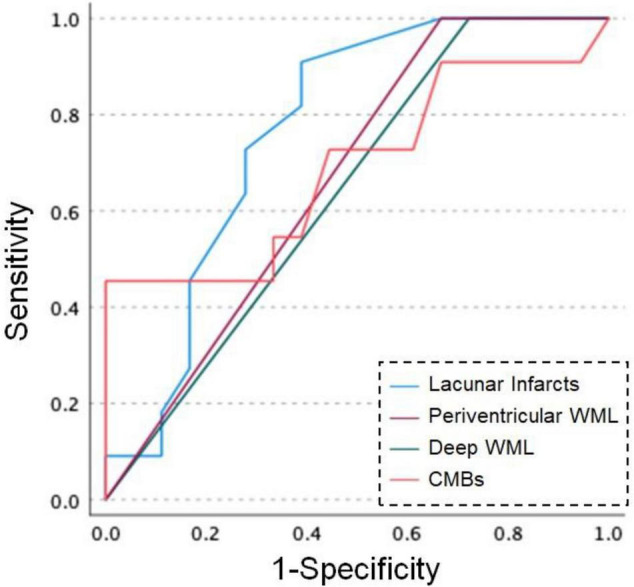
Receiver operating characteristic (ROC) curves for dementia and MRI findings in patients with CADASIL. Blue line: number of lacunar infarcts, purple line: Fazekas scores of periventricular white matter lesion (WML), green line: Fazekas scores of deep WML, orange line: number of cerebral microbleeds (CMBs).

Figure 1 shows the ROC curve analysis of patients with dementia and patients without dementia. For the number of lacunar infarcts, a cut-off score of 5.5 showed 90.9% sensitivity and 61.1% specificity. In periventricular WML, a cut-off score of 2.5 showed 100% sensitivity and 33.3% specificity. In deep WML, a cut-off score of 2.5 showed 100% sensitivity and 27.8% specificity. For the number of CMBs, a cut-off score of 18.5 showed 45.5% sensitivity and 100% specificity.

## Discussion

This study revealed the association between MRI findings and dementia in Japanese patients with CADASIL. The characteristic findings were that CADASIL patients with dementia had higher mRS, severe WML, both periventricular and deep, and a larger number of lacunar infarcts than those without dementia. Moreover, MRI findings of SVD, including CMBs, were associated with a decline in neuropsychological tests. ROC curve analysis showed that 6 or more lacunar infarcts and 19 or more CMBs were associated with dementia in CADASIL patients.

This study revealed the characteristics of patients with CADASIL and dementia. These patients showed severe mRS and lower scores on neuropsychological tests including MoCA-J, MMSE, and FAB, and showed a decline in the completion rate in TMT A and B, as expected. Moreover, both periventricular and deep WML were severe, and the number of lacunar infarcts was significantly higher in patients with dementia. In addition, this study revealed a semi-quantitative association between scores of neuropsychological tests and MRI findings of SVD, including both periventricular and deep WML, lacunar infarcts, and CMBs. The number of lacunar infarcts was associated with decreased MoCA-J and FAB scores, and the number of CMBs was associated with MoCA-J and MMSE scores. Taken together, these SVD imaging markers were related to the decline in neuropsychological tests in several situations. Otherwise, anterior temporal pole lesions did not show any association with neuropsychological tests in this study.

A previous study showed that SVD is a risk factor for both dementia and physical disabilities (Wardlaw et al., 2013), and patients with CADASIL with an mRS score ≥3 demonstrated lower MMSE scores than those with an mRS score < 3 (Yao et al., 2012). Periventricular WML was associated with cognitive decline (Fukuda et al., 1990; van Dijk et al., 2008), while deep WML was associated with a decline in executive function (Brugulat-Serrat et al., 2020). The periventricular WML has a negative correlation with cognitive function in the elderly compared to deep WML (Bolandzadeh et al., 2012). The volume of lacunar infarcts is negatively associated with frontal function in elderly participants (Jokinen et al., 2020), and CMBs are associated with lower MMSE scores in memory clinic patients (Fan et al., 2021). The Rotterdam Study revealed that the presence of more than 4 CMBs was associated with cognitive decline in the general population (Akoudad et al., 2016). Among patients with CADASIL, the number of CMBs gradually increases as the disease progresses, and the number of CMBs in the frontal and temporal lobes and pons is associated with a decline in MMSE score (Chung et al., 2020). Most patients with CADASIL in this study had multiple lacunar infarcts, and periventricular WML could be considered a more sensitive marker for the decline of neuropsychological tests compared to deep WML. Our results demonstrated that a higher number of CMBs was associated with dementia in patients with CADASIL compared to in the general population, which could be attributed to the study background. Moreover, this study clearly supports previous findings that SVD imaging markers may be related to cognitive dysfunction in patients with CADASIL, since these patients have juvenile onset and usually do not suffer from an Alzheimer’s pathology.

Receiver operating characteristic curve analysis between CADASIL patients with and without dementia revealed that the risk of dementia may be associated with 6 or more lacunar infarcts and 19 or more CMBs. In addition, a Fazekas scale score of 3 in both periventricular and deep WML may also be linked to dementia. A previous report revealed that volumes of white matter hyperintensities and lacunar infarcts are predictors of cognitive impairment in SVD (Jokinen et al., 2020). Moreover, the number of CMBs is associated with cognitive impairment in patients with SVD, and a higher number of CMBs might indicate microvascular damage (Nannoni et al., 2021). Our study may enforce these previous studies that higher numbers of lacunar infarcts and CMBs and a severe degree of WML are associated with dementia risk in patients with CADASIL. Moreover, prevention of vascular lesions caused by lifestyle-related diseases may slow the progression of dementia in CADASIL.

Although we revealed the characteristics and MRI findings of the CADASIL patients with dementia, we acknowledge that there are several limitations in our study. First, ePVS and cortical superficial siderosis (cSS) were not evaluated in this study. A previous report revealed that ePVS was detected in temporal pole lesions in patients with CADASIL (Yamamoto et al., 2009). Although cSS has not been found in CADASIL (Wollenweber et al., 2017), SVD scores including ePVS and cSS have been reported to influence neuropsychological tests in memory clinic patients (Matsuda et al., 2021). Moreover, the ePVS burden has also been associated with cognitive impairment (Bown et al., 2021). Moreover, other MRI markers for SVD remain uncertain and may provide additional data in the future. Second, we could not investigate all neuropsychological tests for patients with CADASIL. TMT was conducted in only 17 patients, and we could not compare the time of TMT in this study. Third, we could not longitudinally follow the symptoms, neuropsychological tests, and MRI findings in this study. If we evaluate the changing aspects of neuropsychological tests and MRI findings, the progress of dementia may be clearly revealed in CADASIL. Finally, the diagnosis of CADASIL was made based on the presence of *NOTCH3* mutations in the patient or a third-degree relative. Thirty-three of 44 patients’ gene mutations were assessed in this study, however, a maximum of 11 patients were possibly diagnosed based on their relatives’ gene mutation. However, through this study, we evaluated the relationships between MRI findings and neuropsychological tests and revealed the cut-off scores of MRI SVD markers of CADASIL patients with dementia.

## Conclusion

This study revealed the characteristics of CADASIL patients with dementia and the association between MRI findings and neuropsychological tests. Moreover, the MRI markers for the risk of dementia in patients with CADASIL could have been elucidated. There is a possibility that the prevention of these MRI lesions can reduce the risk of dementia in CADASIL.

## Data Availability Statement

The raw data supporting the conclusions of this article are available from the corresponding author, upon reasonable request. Requests to access the datasets should be directed to AS, a-shindo@med.mie-u.ac.jp.

## Ethics Statement

The studies involving human participants were reviewed and approved by the Ethics Committee of Mie University Graduate School of Medicine (permit number 2918). The patients/participants provided their written informed consent to participate in this study.

## Author Contributions

AT: draft of manuscript and acquisition of data. AS and K-IT: manuscript revision, data acquisition, study concept and design, and analysis. OO, YA, TU, KKIm, KKIt, YM, MT, MI, IM, and TM: acquisition of data and data interpretation. HT: revision of the manuscript, study concept and design, and study supervision. All authors contributed to the article and approved the submitted version.

## Conflict of Interest

The authors declare that the research was conducted in the absence of any commercial or financial relationships that could be construed as a potential conflict of interest.

## Publisher’s Note

All claims expressed in this article are solely those of the authors and do not necessarily represent those of their affiliated organizations, or those of the publisher, the editors and the reviewers. Any product that may be evaluated in this article, or claim that may be made by its manufacturer, is not guaranteed or endorsed by the publisher.
